# A population-based study of social demographic factors, associated diseases, and herpes zoster ophthalmicus in Taiwan

**DOI:** 10.3389/fmed.2025.1532366

**Published:** 2025-03-06

**Authors:** Chia-Yi Lee, Yuh-Shin Chang, Chung-Han Ho, Jhi-Joung Wang, Han-Yi Jan, Po-Han Lee, Ren-Long Jan

**Affiliations:** ^1^Department of Ophthalmology, Chi Mei Medical Centre, Tainan, Taiwan; ^2^School of Medicine, National Sun Yat-sen University, Kaohsiung, Taiwan; ^3^Department of Hospital and Health Care Administration, Chia Nan University of Pharmacy and Science, Tainan, Taiwan; ^4^Department of Medical Research, Chi Mei Medical Centre, Tainan, Taiwan; ^5^School of Medicine, Tzu Chi University, Hualien, Taiwan; ^6^Division of Cardiology, Department of Internal Medicine, Kaohsiung Medical University Hospital, Kaohsiung, Taiwan; ^7^Department of Paediatrics, Chi Mei Medical Centre, Liouying, Tainan, Taiwan

**Keywords:** herpes zoster ophthalmicus, case-controlled study, sociodemographic factors, Taiwan Longitudinal Health Insurance Database, epidemiology

## Abstract

**Introduction:**

Herpes zoster ophthalmicus (HZO) occurs due to the reactivation of latent varicella-zoster virus (VZV) and is characterized by the involvement of the ophthalmic branch of the trigeminal nerve. While this pathophysiology is well-established, the precise mechanisms driving VZV reactivation remain incompletely understood. Furthermore, it is unclear whether individuals with common comorbidities that compromise immune function face an elevated risk of developing HZO. Investigating potential links between HZO and chronic systemic conditions holds significant importance from public health, medical, and scientific perspectives. To address these gaps, we conducted a study to examine the association between HZO development, sociodemographic factors, and systemic comorbidities.

**Materials and methods:**

This nationwide, population-based, retrospective, matched case-controlled study included 52,112 patients with HZO (identified by the International Classification of Diseases, Ninth Revision, Clinical Modification code 053.2 for herpes zoster with ophthalmic complications) from the Taiwan National Health Insurance Research Database. The age-, sex-, and index date-matched control group included 52,112 non-HZO individuals from the Taiwan Longitudinal Health Insurance Database 2000. Sociodemographic factors and associated systemic diseases were examined using univariate logistic regression analyses, and continuous variables were analysed using paired *t*-tests. The odds ratios (ORs) for developing HZO were compared using adjusted logistic regression analysis.

**Results:**

Patients with systemic diseases (hypertension, diabetes, hyperlipidaemia, etc.) had significantly higher ORs for HZO development. Patients whose monthly income was >NT$ 30,000 and patients residing in southern Taiwan had increased odds of developing HZO; however, patients residing in northern Taiwan, metropolitans, or satellite cities, and being public servants (military, civil, teaching staff, etc.) had decreased odds of developing HZO.

**Discussion:**

HZO is strongly associated with hypertension, diabetes mellitus, hyperlipidaemia, coronary artery disease, chronic renal disease, and human immunodeficiency virus infection. These findings emphasise the role of systemic health in HZO risk.

## 1 Introduction

The varicella-zoster virus (VZV, or human herpes virus type 3) causes varicella infection and is found worldwide (1). The virus causes two distinct diseases. Primary infection, also called chickenpox, is caused by the highly contagious and airborne VZV during childhood. After the initial infection, VZV remains in the sensory ganglionic neurones in the trigeminal or dorsal root of the host for a lifelong latent period. In contrast, herpes zoster (HZ), or shingles, is a sporadic neurocutaneous disease caused by reactivation of the latent VZV in the sensory spinal or cerebral ganglia. Subsequently, the virus may travel along neurones to the sensory axons of the skin, resulting in a dermatological rash (1, 2).

Herpes zoster ophthalmicus (HZO) results from the reactivation of latent VZV and is characterized by involvement of the ophthalmic branch of the trigeminal nerve, accounting for 10–25% of all HZ cases (1, 3, 4). Clinically, it presents as a vesicular rash around the eye in the V1 dermatome. Patients with HZO may experience a prodromal phase with symptoms, such as fever, malaise, headache, and ocular pain, before the appearance of the rash. The patients may also report increased eye pressure, tearing, redness, or blurred vision. Pain along the trigeminal nerve can be intense, and chronic pain may develop in the form of postherpetic neuralgia. VZV can infect all layers of the eye, leading to a wide range of ocular manifestations, including conjunctivitis, keratitis, scleritis, uveitis, retinal necrosis, optic neuritis, cranial nerve palsy, and orbital apex syndrome, with outcomes ranging from minimal visual disturbance to permanent vision loss (1, 5–9).

The exact mechanisms underlying the reactivation of latent VZV remain unclear. Historically, a decline in cell-mediated immunity was believed to be a key factor in the reactivation of the VZV and was often cited as the reason why immunosuppressive conditions and advancing age are consistently identified as major risk factors for HZ (5, 10–12). However, recent studies have acknowledged that while HZ is more common and severe in immunocompromised individuals, the majority of HZ cases occur among immunocompetent patients whose risk factors are not well characterized (13–16). A population-based study of 1,669 adult residents in Olmsted County found that 92% of patients with HZ were immunocompetent (13). Additionally, Tran et al. collected data, involving 90 patients, over a span of 4 years, and reported that 78.9% of individuals with HZO in their study were immunocompetent (14). This shift in understanding highlights the need to investigate and characterize the demographic profiles of patients with HZO.

Several previous studies have reported possible associations between HZ and certain chronic conditions (16, 17). Associations between the incidence of HZ and hypertension, diabetes mellitus, hyperlipidaemia, coronary artery disease (CAD), chronic renal disease (CRD), and human immunodeficiency virus infection (HIV) have been recently reported as risk factors for HZ (16, 17). Chronic conditions may contribute to the unexplained burden of HZ (16).

Compared with HZ, HZO has often been underreported in retrospective studies. It remains unclear whether the risk of developing HZO is elevated in patients with common underlying conditions that may impact immune function. Investigating the relationship between HZO and chronic conditions is important for medical, scientific, and public health reasons. To explore the association between sociodemographic factors, various comorbid conditions, and HZO, we conducted a nationwide, population-based case–control study utilizing Taiwan’s National Health Insurance Research Database (NHIRD).

### 1.1 Database

Our cohort study used data from Taiwan’s National Health Insurance Research Database (NHIRD), made available by the National Health Research Institute (NHRI). This database includes encrypted patient identifiers, demographic information (age, sex, and residential area), and admission and discharge dates. It also incorporates the International Classification of Diseases, Ninth Revision, Clinical Modification (ICD-9-CM) codes to cover procedures, diagnoses, prescriptions, and associated costs funded by the NHRI. The Institutional Review Board of the Chi-Mei Medical Center in Tainan has waived the need for ethical approval and informed consent for this study, due to the use of de-identified data.

### 1.2 Selection of patients and variables

**Figure 1:**
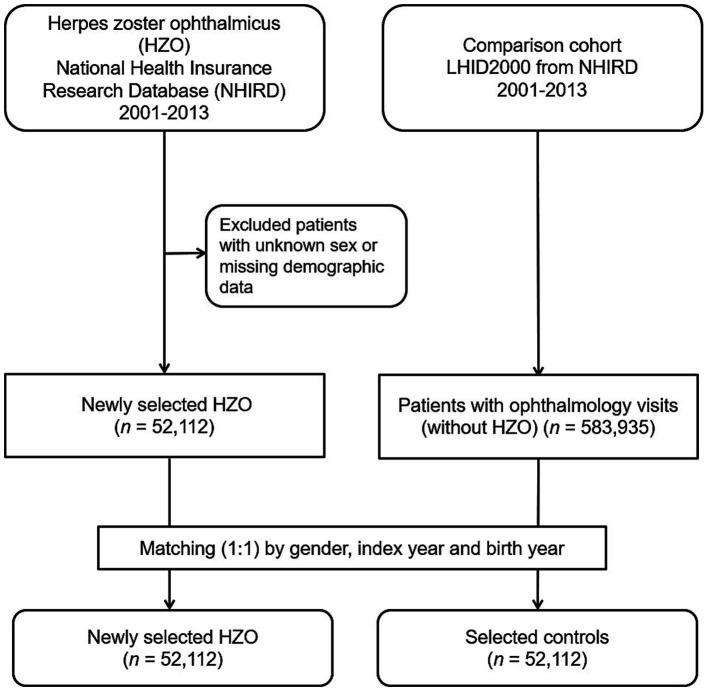
Flowchart detailing the enrollment process for patients diagnosed with herpes zoster ophthalmicus and their control counterparts.

This population-based case–control study included an HZO group (ICD-9-CM code 053.2) and a matched non-HZO control group. Data were collected from 1 January 2001 to 31 December 2013. Figure 1 depicts the study’s flowchart. In total, 52,112 patients diagnosed with HZO were enrolled, and their data were obtained from the NHIRD after excluding patients of unknown sex, those with missing demographic data, and those diagnosed with HZO before 1 January 2001.

For each individual diagnosed with HZO, we randomly selected a control participant (an individual without HZO) from the 2000 Longitudinal Health Insurance Database. This database is a subset of the NHIRD, encompassing comprehensive claims data for one million beneficiaries throughout year 2000. The controls (*n* = 52,112) were matched to the patients with HZO based on age (±30 days), sex, and the index date (the first day of HZO diagnosis). Controls were excluded if they were diagnosed with HZO before the specified index date. To determine the medical comorbidities of the patients with HZO, data regarding comorbid conditions, such as hypertension (ICD-9-CM codes 401–405), diabetes mellitus (ICD-9-CM code 250), hyperlipidaemia (ICD-9-CM code 272), CHF (ICD-9-CM code 428), CAD (ICD-9-CM codes 410–414), CRD (ICD-9-CM codes 582–588 except 584 and 587), HIV infection (ICD-9-CM codes 042 and V08), and post-organ transplantation (ICD-9-CM codes 68035B, 68037B, 68047B, 75020B, and 76021B), were collected. These comorbidities were identified based on the ICD-9-CM codes recorded within the year before the index date and ascertained using three or more ambulatory care claims or inpatient admissions.

### 1.3 Statistical analysis

All statistical analyses were conducted using the SAS software (version 9.4; SAS Institute, Inc., Cary, NC, United States). Demographic characteristics, such as age group, sex, income, geographic region, residential city status, and occupation, were analyzed using McNemar’s test, while continuous variables were evaluated using paired *t*-tests. McNemar’s test was also used to compare comorbid conditions between patients with HZO and controls. Odds ratios (ORs) were calculated using univariate logistic regression analyses, and adjusted ORs for various comorbidities associated with an HZO diagnosis were determined via multivariable logistic regression adjusted for age, sex, and index date. The independent variables in these analyses included sociodemographic factors (income, geographic region, residential city status, and occupation) and all pertinent medical conditions. Statistical significance was set at *p* < 0.05 for all analyses.

## 2 Results

### 2.1 Demographic data

#### 2.1.1 Sociodemographic factors

After excluding ineligible patients, our analysis focused on 52,112 individuals diagnosed with HZO, along with an equal number of age- and sex-matched controls, who utilized medical care services covered by the NHRI from 2001 to 2013. The mean age of patients with HZO and controls was 53.94 years, with a standard deviation of 17.52 (Table 1). Among the 52,112 patients with HZO, 26,655 (51.15%) were male, while 25,457(48.85%) were female.

**Table 1:** Baseline sociodemographic factors and comorbidities of patients with herpes zoster ophthalmicus and age- and sex-matched controls.

	Herpes zoster ophthalmicus *N* = 52,112	Comparison *N* = 52,112	*p*-value
Sociodemographic factors	*n* (%)	*n* (%)	
Age (years; mean ± SD)	53.94 ± 17.52	53.94 ± 17.52	1.0000^a^
Age (years)			
<25	2,819 (5.41)	2,819 (5.41)	1.0000^b^
25–34	5,981 (11.48)	5,981 (11.48)	
35–44	6,562 (12.59)	6,562 (12.59)	
45–54	9,562 (18.35)	9,562 (18.35)	
55–64	11,403 (21.88)	11,403 (21.88)	
≥65	15,785 (30.29)	15,785 (30.29)	
Sex			
Male	26,655 (51.15)	26,655 (51.15)	1.0000^b^
Female	25,457 (48.85)	25,457 (48.85)	
Income			<0.0001^b^
<NT$ 30,000	31,097 (59.67)	32,861 (63.06)	
NT$ 30,000–60,000	16,261 (31.20)	15,450 (29.65)	
NT$ 60,000–90,000	3,472 (6.66)	2,909 (5.58)	
NT$ 90,000–120,000	654 (1.25)	489 (0.94)	
>NT$ 120,000	628 (1.21)	403 (0.77)	
Geographical region of Taiwan			<0.0001^b^
Northern	24,264 (46.56)	25,653 (49.23)	
Central	9,415 (18.07)	10,290 (19.75)	
Southern	17,221 (33.05)	14,585 (27.99)	
Eastern	1,212 (2.33)	1,584 (3.04)	
Residential city status			<0.0001^b^
Metropolis	39,158 (75.14)	36,242 (69.55)	
Satellite	3,044 (5.84)	3,487 (6.69)	
Rural	9,910 (19.02)	12,383 (23.76)	
Occupation			<0.0001^b^
Public servant	26,443(50.74)	25,943 (49.78)	
Farmer	7,741(14.85)	8,606 (16.51)	
Fisherman	1,480(2.84)	954 (1.83)	
Others	16,448(31.56)	16,609 (31.87)	
Comorbid conditions			
Hypertension	15,394 (29.54)	13,830 (26.54)	<0.0001^b^
Diabetes mellitus	7,262 (13.94)	6,421 (12.32)	<0.0001^b^
Hyperlipidaemia	8,211 (15.76)	6,649 (12.76)	<0.0001^b^
Congestive heart failure	1,315 (2.52)	1,083 (2.08)	<0.0001^b^
Coronary artery disease	5,363 (10.29)	4,171 (8.00)	<0.0001^b^
Chronic renal disease	2,115 (4.06)	1,393 (2.67)	<0.0001^b^
Human immunodeficiency virus infection	129 (0.25)	22 (0.04)	<0.0001^b^
Organ transplants	21 (0.04)	15 (0.03)	0.3172^b^

^a^Paired *t*-test; ^b^McNemar’s test; NT$, New Taiwan dollars; SD, standard deviation.

Significant differences emerged in income distribution between patients with HZO and controls (*p* < 0.0001). The predominant monthly income bracket of patients with HZO was below 30,000 New Taiwan dollars (NT$), constituting 59.67% of the cohort (31,097 individuals). Geographic distribution also displayed a notable dissimilarity between the two groups (*p* < 0.0001). Northern Taiwan emerged as the most common region of residence for those diagnosed with HZO, accounting for 46.56% of cases (*n* = 24,264). Examining urban–rural disparities, a substantial majority of patients with HZO resided in metropolitan cities (*n* = 39,158; 75.14%), showing a statistically significant difference when compared with those in rural areas (*n* = 9,910; 19.02%) and satellite cities (*n* = 3,044; 5.84%). Occupational classification further underscored the differences among patients with HZO. Notably, more than half of the 33,190 patients with HZO held positions as public servants, including military, civil, and teaching staff (*n* = 26,443; 50.74%).

#### 2.1.2 Comorbid conditions

Patients with HZO exhibited a significantly higher prevalence of systemic diseases, such as hypertension (*n* = 15,394; 29.54%; *p* < 0.0001), diabetes mellitus, (*n* = 7,262; 13.94%; *p* < 0.0001), hyperlipidaemia (*n* = 8,211; 15.76%; *p* < 0.0001), CHF (*n* = 1,315; 2.52%; *p* < 0.0001), CAD (*n* = 5,363; 10.29%; *p* < 0.0001), CRD (*n* = 2,115; 4.06%; *p* < 0.0001), and HIV infection (*n* = 129; 0.25%; *p* < 0.0001), than controls (Table 1).

### 2.2 Associated risk factors

#### 2.2.1 Sociodemographic factors

We used univariate logistic regression analyses and a multiple logistic regression model (adjusting for age, sex, other sociodemographic factors, and comorbidities) to examine sociodemographic factors, specifically monthly income, geographic region, residential city status, and occupation of patients with HZO and controls. Table 2 presents the results of the study.

**Table 2:** Odds ratios and adjusted odds ratios of various sociodemographic factors and comorbid conditions for herpes zoster ophthalmicus.

	Odds ratio^a^ (95% CI)	*P*-value	Adjusted odds ratio^b^ (95% CI)	*P*-value
Sociodemographic factors				
**Income**				
<NT$ 30,000	1.00		1.00	
NT$ 30,000–60,000	1.15 (1.12–1.18)	<0.0001	1.19 (1.15–1.22)	<0.0001
NT$ 60,000–90,000	1.32 (1.25–1.40)	<0.0001	1.39 (1.31–1.47)	<0.0001
NT$ 90,000–120,000	1.49 (1.32–1.68)	<0.0001	1.59 (1.41–1.79)	<0.0001
>NT$ 120,000	1.74 (1.53–1.98)	<0.0001	1.87 (1.64–2.13)	<0.0001
Geographical region of Taiwan				
Northern	1.23 (1.14–1.33)	<0.0001	0.86 (0.79–0.94)	0.0005
Central	1.20 (1.10–1.30)	<0.0001	1.00 (0.92–1.09)	0.9924
Southern	1.54 (1.43–1.67)	<0.0001	1.21 (1.11–1.31)	<0.0001
Eastern	1.00		1.00	
Residential city status				
Metropolis	0.81 (0.77–0.85)	<0.0001	0.84 (0.80–0.88)	<0.0001
Satellite	0.74 (0.71–0.76)	<0.0001	0.67 (0.65–0.70)	<0.0001
Rural	1.00		1.00	
Occupation				
Public servant	1.05 (1.02–1.08)	0.0025	0.96 (0.93–0.99)	0.0058
Farmer	0.89 (0.85–0.92)	<0.0001	0.97 (0.93–1.01)	0.1750
Fisherman	1.58 (1.45–1.72)	<0.0001	1.59 (1.16–1.74)	<0.0001
Others	1.00		1.00.	
Comorbid conditions				
Hypertension	1.21 (1.17–1.25)	<0.0001	1.10 (1.06–1.14)	<0.0001
Diabetes mellitus	1.16 (1.12–1.21)	<0.0001	1.04 (1.00–1.08)	0.0489
Hyperlipidaemia	1.30 (1.25–1.35)	<0.0001	1.20 (1.16–1.25)	<0.0001
Congestive heart failure	1.23 (1.13–1.34)	<0.0001	1.09 (1.00–1.19)	0.0536
Coronary artery disease	1.35 (1.29–1.41)	<0.0001	1.24 (1.18–1.30)	<0.0001
Chronic renal disease	1.56 (1.45–1.67)	<0.0001	1.44 (1.34–1.55)	<0.0001
Human immunodeficiency virus infection	6.10 (3.84–9.67)	<0.0001	6.44 (4.05–10.25)	<0.0001
Organ transplants	1.40 (0.72–2.72)	0.3196	1.33 (0.68–2.61)	0.4096

^aOdds ratios were obtained from univariate logistic regression analyses; bAdjusted odds ratios were calculated from a multivariable logistic regression model that was conditioned on age group, sex, and year of index date.^

NT$, New Taiwan dollars; CI, confidence interval.

Patients with monthly incomes such as NT$ 30,000–60,000, NT$ 60,000–90,000, NT$ 90,000–120,000, and >NT$ 120,000 exhibited increased odds of developing HZO compared to those with an income < NT$ 30,000, even after adjusting for confounding factors. Concerning geographic location, patients residing in southern Taiwan demonstrated a significantly higher prevalence of HZO than those residing in eastern Taiwan, and this remained a significant risk factor following a conditional logistic regression analysis (adjusted OR: 1.21, 95% CI: 1.11–1.31, *p* < 0.0001). Notably, northern Taiwan showed a significantly reduced risk of developing HZO even after adjusting for confounding factors (adjusted OR: 0.86, 95% CI: 0.79–0.94, *p* = 0.0005). Examining residential city status, patients living in metropolitan and satellite cities exhibited a significantly lower risk of developing HZO than those living in rural areas, even after conducting a conditional logistic regression analysis (adjusted OR: 0.84, 95% CI: 0.80–0.88, *p* < 0.0001; adjusted OR: 0.67, 95% CI: 0.65–0.70, *p* < 0.0001; respectively). Regarding occupation, public servants exhibited a significantly lower prevalence of HZO following a multiple logistic regression analysis (adjusted OR: 0.96, 95% CI: 0.93–0.99, *p* = 0.0058). Individuals engaged in fishing faced a significant risk of developing HZO, an independent risk factor even after adjusting for confounders (adjusted OR: 1.59, 95% CI: 1.16–1.74, *p* < 0.0001), as indicated in Table 2.

#### 2.2.2 Comorbid conditions

Univariate and multiple logistic regression analyses were performed to explore several potential comorbidities (Table 2). Patients with systemic diseases, such as hypertension, diabetes mellitus, hyperlipidaemia, CHF, CAD, and CRD, exhibited significantly higher ORs for receiving an HZO diagnosis (OR: 1.21, 95% CI: 1.17–1.25, *p* < 0.0001; OR: 1.16, 95% CI: 1.12-–1.21, *p* < 0.0001; OR: 1.30, 95% CI: 1.25–1.35, *p* < 0.0001; OR: 1.23, 95% CI: 1.13–1.34, *p* < 0.0001; OR: 1.35, 95% CI: 1.29–1.41, *p* < 0.0001; OR: 1.56, 95% CI: 1.45–1.67, *p* < 0.0001, respectively) than those without systemic diseases. Except for CHF, these associations, including hypertension, diabetes mellitus, hyperlipidaemia, CAD, and CRD, remained significant even after conducting conditional logistic regression analyses (adjusted OR: 1.10, 95% CI: 1.06–1.14, *p* < 0.0001; adjusted OR: 1.04, 95% CI: 1.00–1.08, *p* = 0.0489; adjusted OR: 1.20, 95% CI: 1.16–1.25, *p* < 0.0001; adjusted OR: 1.24, 95% CI: 1.18–1.30, *p* < 0.0001; adjusted OR: 1.44, 95% CI: 1.34–1.55, *p* < 0.0001, respectively). Patients with HIV infection also had significantly increased odds of an HZO diagnosis both before (OR: 6.10, 95% CI: 3.84–9.67, *p* < 0.0001) and after adjustment for other confounders (adjusted OR: 6.44, 95% CI: 4.05–10.25, *p* < 0.0001). Since hospitalization records for HZO patients can serve as a proxy indicator of HZO severity, we conducted a sensitivity analysis on the odds ratios for various sociodemographic factors and comorbid conditions among these patients, as detailed in the Supplementary Tables S1, S2. Our analysis identified critical sociodemographic and clinical risk factors for HZO, which we further contextualize below in relation to existing literature and clinical practice.

## 3 Discussion

To the best of our knowledge, this study is the most extensive nationwide, population-based, case–controlled investigation assessing the relationship between sociodemographic factors, prevalent comorbid conditions, and HZO. Our analyses revealed that HZO was more common in male individuals, with a 51.15% predominance. In addition, some comorbidities, including hypertension, diabetes mellitus, hyperlipidaemia, CAD, CRD, and HIV infection, significantly influenced the risk of developing HZO. A high socioeconomic status was associated with higher odds of developing HZO. However, patients living in the metropolis or satellite cities had considerably lower odds of developing HZO than those living in rural areas.

Of the 52,112 patients with HZO, the average age at diagnosis was 53.94 years (SD: 17.52), which is in accordance with the results of several retrospective cohort studies. The Colombian Ocular Infectious Epidemiology Study, which enrolled 2,378 patients with HZO from 2015 to 2019, found that the most frequent age of diagnosis was among the quinquennial group between 55 and 59 years (18). Davies et al. reported that the mean age of patients with acute HZO who presented at the Massachusetts Eye and Ear Infirmary was 55.8 years in 2013 (19). The study also observed a trend of decreasing age at the time of HZO diagnosis over the years (61.2 years in 2007 vs. 55.8 years in 2013, *p* = 0.0119). Chan et al. (20) analyzed patients with HZO at the University of Oklahoma and divided the cases into two cohorts: individuals diagnosed with HZO between 1996 and 2004 (*n* = 130) and those diagnosed between 2005 and 2012 (*n* = 270). The findings indicated a notable reduction in the average age at HZO onset in the latter cohort (65.5 years vs. 58.9 years) (20). One hypothesis for the trend of decreasing age at the time of HZO presentation that has been widely debated is that the introduction of a universal childhood vaccination program for varicella may have impacted HZ epidemiology (19, 20). In 1995, the United States was the first nation to implement a one-dose varicella vaccination mandate for children. Following this, in 1998, the capital city of Taiwan introduced a similar one-dose varicella vaccination requirement for children. This program was later expanded nationwide in Taiwan in 2004. These developments may explain the alignment of their results with our findings.

In our study, patients with HZO showed a slight male predominance (51.15%), which differs from the female predominance typically reported in Western literature (4, 18, 21, 22). However, studies from India, Pakistan, and Nepal have reported a male predominance in HZ cases (23–25). A comprehensive review of the literature dating back to 1865 concluded that HZ does not show a clear preference for either sex (1). Similarly, a review from India has suggested that HZO is unlikely to be sex-specific (26). The sex distribution of HZO remains underexplored in the current body of literature.

Our study found that individuals with a higher socioeconomic status exhibited increased odds of developing HZO. The activity or risk of HZO is influenced by various socioeconomic factors such as income, educational attainment, and living conditions. Those with higher income are likely to have greater awareness and understanding of ocular disorders, causing them to seek prompt medical attention for ophthalmic issues. Thus, socioeconomic status may play a significant role in the timely receipt of treatment and follow-up care in patients with HZO. Importantly, patients with HZO in Taiwan did not seem to experience income barriers related to the disease.

Regarding geographic location, patients in southern Taiwan had a significantly higher prevalence of HZO than those in eastern Taiwan, and this difference remained a significant risk factor after conditional logistic regression analysis. Eastern Taiwan may face challenges in accessing public health programs, particularly in remote or underserved communities. Eastern Taiwan is predominantly mountainous, which may have contributed to the relatively low incidence of HZO observed in our study. This lower rate could be indicative of difficulties such as limited access to medical care, hesitance to seek ophthalmological consultations, and a shortage of specialists available to diagnose and manage HZO. These issues may be more evident in this region than in other parts of Taiwan. In contrast, southern Taiwan may benefit from a better healthcare infrastructure. In addition, the warmer and more humid climate in southern Taiwan may have increased the risk of HZO. A time-series analysis conducted in China reported 43,547 cases of HZ, emphasizing that high temperatures and elevated relative humidity significantly affect the incidence of HZ (27).

Furthermore, we observed a notably lower OR for HZO among individuals residing in northern Taiwan. Northern Taiwan, particularly the Taipei region, is more urbanized, with better healthcare infrastructure and access to preventive medical care. Variations also exist in public health initiatives across different regions of Taiwan, and the availability of more resources in northern Taiwan may result in increased awareness and prevention efforts for HZO. This can contribute to higher vaccination rates (e.g., shingles vaccines) due to enhanced healthcare awareness and better management of conditions that predispose individuals to HZO. Additionally, this might explain the significantly lower prevalence of HZO observed among public servants and residents of metropolitan areas or satellite cities, as these individuals may benefit from more effective immunization programs (e.g., higher uptake of shingles vaccinations) and exhibit more health-conscious behaviors as a result of public health campaigns.

Some studies have reported that certain factors, such as physical or emotional stress and fatigue, may precipitate HZ outbreaks (28). Kawai and Yawn (29) also reported in a meta-analysis that psychological stress may predispose an individual to developing HZ. Fishermen often work long hours under physically demanding conditions, which can lead to chronic stress and fatigue. They frequently work irregular hours, including night shifts and spend several days or weeks at sea. Our findings indicate that fishermen have a higher risk of HZO, likely due to a combination of factors including chronic occupational stress, exposure to harsh environmental conditions, and disrupted sleep patterns. Furthermore, we performed a sensitivity analysis restricted to hospitalized patients, which revealed a reduced odds ratio (OR) for HZO among fishermen compared to the general population. This paradoxical shift (from OR > 1 to OR < 1) may be attributable to two key factors. First, fishermen may encounter systemic barriers to hospitalization—such as remote work environments or delayed care-seeking—resulting in only the most severe HZO cases being admitted. In contrast, non-fishermen with milder HZO symptoms might access hospitalization more readily due to better healthcare access, thereby diluting the observed risk within the hospitalized cohort. Second, fishermen could face higher baseline morbidity or mortality from non-HZO causes (e.g., occupational injuries, cardiovascular diseases), disproportionately reducing their likelihood of hospitalization specifically for HZO. This competing risk dynamic may obscure the true association between occupational status and HZO severity in hospitalized populations.

In our study, patients diagnosed with hypertension showed a significantly higher OR for developing HZO (adjusted OR: 1.10, 95% CI: 1.06–1.14, *p* < 0.0001). A retrospective cohort study of 658 patients diagnosed with HZO in 2003 and 2004 similarly reported a strong association between HZO and pre-existing hypertension (*p* < 0.0001) (30). In addition, a large-scale multicentre study using a medical database in Japan found that hypertension and dyslipidaemia (adjusted OR: 1.28, 95% CI: 1.18–1.40, *p* < 0.05; adjusted OR: 1.11, 95% CI: 1.004–1.22, *p* < 0.05, respectively) were significantly linked to an increased risk of HZ in patients with rheumatoid arthritis (31). However, Joesoef et al. (16) investigated whether common chronic conditions affect the development of HZ in individuals aged 20–64 years and found that hypertension was not a significant risk factor for HZ. Several cohort studies and a meta-analysis conducted in 2017 reviewing comorbidities as risk factors for HZ did not identify hypertension as a risk factor (29, 32). To date, no comprehensive study has specifically evaluated the relationship between hypertension and the risk of HZO in the general population.

Patients with diabetes mellitus demonstrated a significantly increased risk of developing HZO in our study (adjusted OR: 1.04, 95% CI: 1.00–1.08, *p* < 0.0489). A review by Kaiserman et al. (33) highlighted a notably higher incidence of herpes eye disease in patients with diabetes than in those without diabetes. Similarly, Lin et al. (30) found that patients with HZO were more likely to have preexisting diabetes (*p* < 0.0001). Additional studies have highlighted the link between HZ and diabetes. A meta-analysis reviewing 88 studies showed that diabetes significantly increased the risk of HZ compared to controls (risk ratio: 1.24; 95% CI: 1.14–1.35) (34). A population-based cohort study in Taiwan, which examined the connection between diabetes and HZ using real-world data, also identified diabetes as a prominent risk factor for HZ infection (adjusted hazard ratio: 1.17, 95% CI: 1.10–1.23) (35). A synthesis of 11 studies found that the risk of HZ was significantly higher in patients with diabetes than that in controls, with the associated risk ranging from 1.06 to 2.38 across the studies (*p* < 0.05) (36). Additionally, a retrospective cohort study by Guignard et al. (37) using the Integrated Health Care Information Services database from 1997 to 2006 indicated that type II diabetes is linked to an increased risk of developing HZ. This risk was particularly evident in adults aged 65 years and older, but moderately elevated in those under 65 years of age (37). The increased risk may be attributed to the weakened cell-mediated immunity, persistent low-grade inflammation, and microvascular damage commonly observed in diabetes. A study in Japan utilized an interferon-*γ* enzyme-linked immunospot assay to assess VZV-specific cell-mediated immunity in patients with and without diabetes. The findings suggest that the increased risk of HZ among individuals with diabetes may be linked to a reduction in VZV-specific cell-mediated immunity. This impaired immune response likely contributes to the increased susceptibility to HZ infection (38–42).

To date, several studies have discussed cardiovascular diseases as risk factors for HZ infection; however, the mechanism underlying this relationship has not been fully elucidated (16, 34, 43). In our study, hyperlipidaemia and CAD were identified as potential risk factors for HZO (adjusted OR: 1.20, 95% CI: 1.16–1.25, *p* < 0.0001; adjusted OR: 1.24, 95% CI: 1.18–1.30, *p* < 0.0001, respectively). We identified only one study that specifically examined the association between cardiovascular diseases and HZO. Majority of the existing studies primarily focused on HZ infection rather than HZO, leaving a gap in the understanding of cardiovascular risk factors specifically related to HZO. Another study in Taiwan also highlighted a strong association between HZO, hyperlipidaemia, and CAD (*p* < 0.0001) (30). A Taiwanese cohort study involving 67,113 participants in the CAD group revealed that the overall incidence of HZ was higher in the CAD group than in the non-CAD group, after adjusting for confounding factors such as sex, age, and comorbidities (HR: 1.21; 95% CI: 1.14–1.27) (44). Horev et al. (45) identified dyslipidaemia and prior myocardial infarction as significant risk factors for HZ by analyzing an extensive computerized health maintenance organization database. Similarly, a nationwide Korean population-based case–control cohort study found that patients with a history of myocardial infarction had a higher risk of hospitalization for HZ (HR: 1.625; 95% CI: 1.144–2.308). Chronic inflammation, age-related immune changes, endothelial dysfunction, and psychosocial stress have been suggested as potential contributors to this observed association (30, 46, 47).

In our study, CHF was not a significant risk factor for the development of HZO (adjusted OR: 1.09, 95% CI: 1.00–1.19, *p* = 0.0536). We did not find any studies that specifically investigated the relationship between CHF and HZO. Similar to other comorbidities, the majority of existing studies focused on HZ infection rather than HZO. Wu et al. (48) conducted a nationwide population-based cohort study and identified a significant correlation between CHF and an increased risk of HZ during the 1-year follow-up period (adjusted HR: 2.07; 95% CI: 1.54–2.78, *p* < 0.001). In addition, a case–control study in Korea suggested a reciprocal relationship between severe HZ requiring hospitalization and CHF, showing that patients with CHF were at a higher risk of HZ-related hospitalization (HR: 1.485, 95% CI: 1.041–2.117) (49). However, a meta-analysis by Kawai and Yawn (29), reviewing comorbidities as risk factors for HZ did not highlight CHF as a significant factor. A commonly accepted explanation for the increased risk of HZ in patients with CHF is that these individuals are exposed to numerous physiological and psychological stressors that may make them more susceptible to HZ reactivation (50, 51). However, CHF diagnosis often relies on clinical evaluation rather than standardized serologic testing. Therefore, diagnoses based on ICD-9-CM codes may be inaccurate. Furthermore, the use of ICD-9-CM codes may not accurately capture the severity of CHF, which could influence the assessment of its association with HZ reactivation and potentially affect our findings. Additionally, we conducted a sensitivity analysis focusing solely on hospitalized patients. In this analysis, among all comorbid conditions, only hyperlipidemia ceased to be a risk factor. This change may be because hospitalized HZO patients are admitted due to more severe conditions, which may not be related to hyperlipidemia but could mask or dilute its independent effect. Outpatient cases may include milder or early-stage HZO patients, where the impact of hyperlipidemia as a chronic metabolic factor is more observable.

In our study, CRD was identified as a significant risk factor for HZO, with an adjusted OR of 1.44 (95% CI: 1.34–1.55, *p* < 0.0001). While no studies have specifically investigated the relationship between CRD and HZO, substantial evidence suggests that CRD is an important risk factor for HZ in general (17, 29, 34, 52–57). Hata et al. (17), in a retrospective hospital-based cohort study of 55,492 patients, reported that renal failure significantly increases the risk of HZ events (adjusted HR: 2.14, 95% CI: 1.65–2.79). Similarly, Wu et al. (53) identified 13,321 patients with CRD and found that CRD was independently associated with a higher risk of HZ (HR: 1.60, 95% CI: 1.41–1.81). Lai et al. (57) also demonstrated a 1.4-fold increase in the incidence of HZ in pre-dialysis patients with CRD compared to that in patients without CRD (8.76 vs. 6.27 per 1,000 person-years, 95% CI: 1.27–1.54; *p* < 0.001). These findings underscore the fact that CRD is a significant contributor to the risk of HZ, which may extend to ophthalmic manifestations such as HZO. Patients with CRD have an increased risk of HZ infection, likely because of various factors that compromise their immune systems. A key factor is the loss of immune-related proteins, which weakens the immune response in these patients (58). Additionally, the frequent exposure to viral and bacterial pathogens during regular dialysis treatments further increases their susceptibility (59). The correlation between immune deficiency and the incidence of infections in patients with CRD is well established, with both innate and adaptive immune systems showing significant impairments, leading to a higher risk of infectious complications (60, 61).

Clinically, many believe that patients with HIV have a greater prevalence of HZ or HZO infections than the general population. In our study, HIV was identified as a significant risk factor for HZO with an adjusted OR of 6.44 (95% CI: 4.05–10.25, *p* < 0.0001) (62–65). Studies have indicated an association between HIV and HZO: HIV seroprevalence in patients with HZO varies from 40 to 100% in Africa (66). Gupta et al. (67) reported the clinical profile of HZO in adults younger than 40 years and found that 44.4% of the subjects were positive for HIV. HIV primarily targets CD4+ T cells, which are crucial for maintaining control over latent viral infections, including the VZV. As the immune system becomes progressively weakened because of HIV infection, the body loses its ability to control latent VZV, leading to its reactivation as HZ (1).

Our study has several strengths. To date, this is the largest investigation to comprehensively explore the relationship between sociodemographic factors, common comorbidities, and HZO. By analyzing a wide range of sociodemographic variables, such as income, occupation, and geographic distribution, this study offers a broad understanding of HZO incidence in the general population. The use of a nationwide population-based dataset minimized selection bias from referral centers, and reliance on electronically recorded data from the NHIRD database eliminated recall bias. With 13 years of longitudinal data, this case–control study allowed for a thorough examination of sociodemographic factors and chronic diseases in both patients with HZO and controls. Moreover, adjusting for confounding variables ensured the reliability of our findings.

However, this study had some limitations. First, the use of ICD-9-CM codes to diagnose HZO and comorbidities may have led to misclassification of the disease. A lack of access to clinical records also prevented the confirmatory diagnosis of HZO in patients and controls. Additionally, as the study was conducted in a Taiwanese population, the generalisability of these results may be limited in other regions or populations with different sociodemographic characteristics. Although we controlled for several confounders, unmeasured lifestyle factors—such as smoking status, body mass index, or psychological stress—that may affect immune function could still have influenced the observed associations. Furthermore, medical history tracing was only possible in 1996, leaving a gap in confirming prior HZO diagnoses. Future studies should incorporate clinical information, questionnaires, lifestyle factors, and other sociodemographic and pathophysiological factors to address these limitations. Finally, in our retrospective case–control study, it is challenging to determine whether the exposure preceded the outcome or vice versa, which complicates causal inference and obscures the temporal relationship. Additionally, accurately quantifying exposure levels after the outcome has occurred is complex and typically less precise than in cohort studies. Moreover, unlike cohort studies, case–control studies do not allow for the calculation of disease incidence rates, as the total population at risk at the beginning of the study period is unknown. In the future research, cohort studies should be used to measure incidence rates, which are essential for assessing the public health impact of a condition.

In conclusion, this nationwide study reaffirms that herpes zoster ophthalmicus is strongly associated with systemic comorbidities—hypertension, diabetes, hyperlipidemia, CAD, CRD, and HIV—and sociodemographic factors such as higher income, southern residency, and occupations like fishing. Notably, urbanized regions and public servants exhibited reduced risk, likely due to better healthcare access and preventive measures. These findings directly address our research objective of delineating HZO risk profiles, offering actionable insights for clinicians and policymakers. Key recommendations include prioritizing high-risk groups for vaccination, enhancing rural ophthalmologic infrastructure, and integrating HZO screenings into chronic disease management protocols. By aligning public health strategies with these evidence-based risk factors, Taiwan—and similar populations—can reduce the burden of HZO and its vision-threatening sequelae.

## Data Availability

The original contributions presented in the study are included in the article/Supplementary material, further inquiries can be directed to the corresponding author.
