# Supplementary material for: A population-based study of social demographic factors, associated diseases, and herpes zoster ophthalmicus in Taiwan
Source: Front Med (Lausanne). 2025 Mar 6;12:1532366. doi: 10.3389/fmed.2025.1532366 (PMC11922945; doi:10.3389/fmed.2025.1532366)
Supplement: Supplementary file 1 [file Table_1.docx]

**# Supplemental table 1 (Table 1S)**. Number of herpes zoster ophthalmicus (HZO) outpatient visits and hospitalization records with HZO

|  | HZO |
| --- | --- |
| Number of outpatient visits for HZO, mean±SD | 2.82±5.25 |
| Hospitalization records with HZO, n (%) | 4284 (8.22) |

**# Supplemental Table 2 (Table 2S)**. Sensitivity analysis of the odds ratios for various sociodemographic factors and comorbid conditions among patients with herpes zoster ophthalmicus (HZO) hospitalization records

|  | Adjusted odds ratio  (95% CI) | P value |
| --- | --- | --- |
| Sociodemographic factors |  |  |
| Income |  |  |
| <NT$ 30,000 | 1.00 |  |
| NT$ 30,000–60,000 | 1.05 (0.97-1.14) | 0.2372 |
| NT$ 60,000–90,000 | 1.21 (1.05-1.40) | 0.0108 |
| NT$ 90,000–120,000 | 1.42 (1.05-1.91) | 0.0226 |
| >NT$ 120,000 | 1.12 (0.78-1.60) | 0.5498 |
| Geographical region of Taiwan |  |  |
| Northern | 0.72 (0.61-0.85) | 0.0002 |
| Central | 0.61 (0.51-0.72) | <0.0001 |
| Southern | 0.61 (0.51-0.72) | <0.0001 |
| Eastern | 1.00 |  |
| Residential city status |  |  |
| Metropolis | 0.99 (0.91-1.09) | 0.8801 |
| Satellite | 0.72 (0.61-0.84) | <0.0001 |
| Rural | 1.00 |  |
| Occupation |  |  |
| Public servant | 0.91 (0.84-0.99) | 0.0301 |
| Farmer | 0.92 (0.83-1.01) | 0.0886 |
| Fisherman | 0.75 (0.57-0.98) | 0.0370 |
| Others | 1.00 |  |
| Comorbid conditions |  |  |
| Hypertension | 1.12 (1.04-1.21) | 0.0034 |
| Diabetes mellitus | 1.17 (1.07-1.28) | 0.0005 |
| Hyperlipidaemia | 1.03 (0.94-1.13) | 0.5117 |
| Congestive heart failure | 1.46 (1.25-1.72) | <0.0001 |
| Coronary artery disease | 1.24 (1.12-1.38) | <0.0001 |
| Chronic renal disease | 1.92 (1.68-2.20) | <0.0001 |
| Human immunodeficiency virus infection | 20.78 (11.90-36.27) | <0.0001 |
| Organ transplants | 1.42 (0.32-6.27) | 0.6417 |
